# IGFBP-3 Interacts with the Vitamin D Receptor in Insulin Signaling Associated with Obesity in Visceral Adipose Tissue

**DOI:** 10.3390/ijms18112349

**Published:** 2017-11-07

**Authors:** Inmaculada Moreno-Santos, Daniel Castellano-Castillo, María Fernanda Lara, Jose Carlos Fernandez-Garcia, Francisco Jose Tinahones, Manuel Macias-Gonzalez

**Affiliations:** 1 Unidad de Gestión Clínica Endocrinología y Nutrición, Instituto de Investigación Biomédica de Málaga (IBIMA), Complejo Hospitalario de Málaga (Virgen de la Victoria)/Universidad de Málaga, 29010 Málaga, Spain; morenosantos.inma@gmail.com (I.M.-S.); danie__cc@hotmail.com (D.C.-C.); josecarlosfdezgarcia@hotmail.com (J.C.F.-G.); 2 CIBER Pathophysiology of Obesity and Nutrition (CB06/03), 29010 Málaga, Spain; 3 Department of Urology, Complejo Hospitalario de Málaga (Virgen de la Victoria)/Universidad de Málaga, 29010 Málaga, Spain; fer76lc@hotmail.com

**Keywords:** vitamin D, VDR, IGFBP, insulin resistance, morbid obesity and adipose

## Abstract

Adipose tissue has traditionally only been considered as an energy storage organ. Nevertheless, the importance of this tissue in systemic physiology and, especially, in systemic inflammation has been highlighted in recent years. Adipose tissue expresses proteins related to vitamin D (VD) metabolism, and it has been proposed that it can act as a VD storage tissue. The active form of VD, 1,25-dihydroxyvitamin D3 (1,25(OH)_2_D_3_), is able to modify adipocyte and adipose tissue physiology via the VD receptor (VDR), decreasing the expression of pro-inflammatory cytokines in adipose tissue. Moreover, VD deficiency and VDR has been reported to be associated with obesity and diabetes. However, the results of the different studies are not conclusive. Insulin growth binding proteins (IGFBPs) have been identified in adipose tissue, but their roles are poorly understood. Therefore, the objective of this study was to analyze the plasma levels of VD and the gene expression of VDR in the adipose tissue of subjects with morbid obesity (MO) and with different degrees of insulin resistance (IR), as well as the functionality of direct interaction between IGFBP-3 and VDR, which could explain its inhibitory role in adipogenesis. Our results show a novel role of the VD system in the regulation and activation of IGFBP-3 in visceral adipose tissue (VAT) of patients with MO, as a new and alternative mechanism proposed in the insulin signaling associated with obesity.

## 1. Introduction

Adipose tissue has long been identified as the main storage site for vitamin D (VD) [1]. In addition, it has been shown that VD regulates adipogenic gene expression, and is active in adipocytes at all levels. VD reduces the release of cytokines and the inflammation of the visceral adipose tissue (VAT) through the inhibition of nuclear factor kappa-light-chain-enhancer of activated B cells (NF-κB) signaling. Obesity is associated with VD deficiency [1]. Furthermore, recent studies have shown that the vitamin D receptor (VDR) is expressed in adipocytes. VDR is a transcription factor which binds to specific VD response elements (VDREs) within the regulatory regions of its primary target genes [2]. Retinoid x receptor α (RXRa) is also a ligand-dependent transcription factor, whose functions by dimerizing with itself or with several other type II nuclear receptor (NR) family members, including VDR [3]. The protein-DNA complex of a VDR-RXR heterodimer binding to a VDRE, therefore, can be considered as a molecular switch for primary 1,25(OH)_2_D_3_ responding genes [4].

Mitogens, such as the insulin like growth factors (IGFs) and IGF binding proteins (IGFPBs), have been reported to be regulated by 1,25(OH)_2_D_3_ [5], of which the *IGFBP-3* gene is the most prominent [6]. IGF-I is a well-established inducer of preadipocyte differentiation [7]. In adults, IGFBP-3 transports most of the IGF-I and IGF-II, which are bound in ternary complexes. However, the ability of IGFBP-3 to inhibit 3T3-L1 preadipocyte differentiation independently of IGF binding suggests that the IGF receptor independent mechanism is mediated [8].

The *IGFBP-3* gene is a transcriptional target of the VDR [9], and the *IGFBP-3* gene has two VDRE sequences, one at position 400 and the other VDRE at position 3350 relative to the transcription start site (TSS) [10]. IGFBP-3 has also been reported to inhibit VDR-dependent transcriptional activity [11]. Moreover, the interaction between IGFBP-3 and RXRa is of particular interest in the context of adipocyte differentiation because VDR is recognized as an obligate dimerization of RXRa [12]. 

In vivo and in vitro studies indicate a major role of VDR in adipocyte biology, suggesting that VDR inhibits adipogenesis [13]. The first evidence of an inhibitory role of IGFBP-3 on differentiated adipocytes was that IGFBP-3 inhibits insulin-stimulated glucose uptake [14]. These studies altogether indicate a strong inhibitory effect of IGFBP-3 on adipogenesis, at least in some experimental systems, and suggest that the induction of endogenous IGFBP3 during fat cell differentiation might have a negative feedback role. 

Interestingly, IGFBP-3 stimulated glycerol-3-phosphate dehydrogenase (G3PD) activity during differentiation of visceral and subcutaneous preadipocytes isolated from adipose tissue of children, suggesting enhanced differentiation [15], which is in contrast to the inhibitory effect observed on 3T3-L1 preadipocyte differentiation [16]. As recently reviewed, IGFBP-3 can be either inhibitory or stimulatory to the differentiation of a variety of the cell types under different conditions [17], but the factors that account for this divergence between its effects in 3T3-L1 cells and preadipocytes from adipose tissue of children are not yet understood. 

Genetic polymorphisms that affect *IGFBP-3* and *VDR* are hypothesized to influence cancer risk through a mechanism of altered IGF signaling. One mechanism for the effect VD has on cell proliferation is modulation of IGF signaling via VDR-mediated stimulation of IGFBP-3 synthesis [18]. Therefore, inherited polymorphisms affecting VDR can be hypothesized to affect IGF signaling and, in turn, growth, obesity, and obesity-related diseases. Finally, binding proteins IGFBPs and structurally-related proteins have been identified in adipose tissue although their roles are poorly understood [19].

Recent studies have highlighted that obesity activates the NF-κB, which play an important role in inflammation-induced insulin resistance (IR) [20]. It has been demonstrated that NF-κB antagonizes the transcriptional regulatory activity of VDR in adipocytes [21]. Therefore, the balance between VDR, NF-κB, and possibly other DNA-binding proteins is likely important for adipocyte function and response to insulin [21]. This places the VDR-responsive members of the *IGFBP* genes family in the position of critical interfaces in the response to this nuclear receptor and, hence their ligands, dietary components, emphasizing their role as biomarkers in obesity, as well as in IR. 

To date, there are no published clinical studies comparing protein levels or gene expression of *IGFBPs* and related proteins in VAT or between the insulin-sensitive and insulin-resistant associated with obesity. It has also been demonstrated that 1,25(OH)_2_D_3_ could directly modulate NF-κB activation [22], suggesting that targeting these genes may likely be an important mechanism of inhibitory actions of VD. Thus, to evaluate the molecular mechanism linking obesity and IR we aimed to examine the interplay between *IGFBPs* and *VDR*. Similarly, we studied the presence and activation of NF-κB in the VAT from morbidly obese (MO) subjects associated with different degrees of IR.

## 2. Results

### 2.1. Baseline Clinical Characteristics of the Participants

Table 1 shows the biochemical and anthropometric characteristics of study groups. There were no differences in age or gender among all the groups. Weight and the related anthropometric data were, as expected, significantly greater in MO patients. HIR-MO subjects presented higher levels of glucose, triglycerides, insulin, and HOMA-IR, in comparison with LIR-MO subjects. Serum 25-OH-VD levels decreased significantly as the BMI and the insulin resistance increased. 

### 2.2. Association between Serum 25-OH-VD and VAT VDR mRNA Expression with Different Clinical Variables in All the Patients

Table 2 shows serum 25-OH-VD correlated negatively with all the anthropometric parameters related with obesity (weight, BMI, and waist circumference), and with glucose and insulin levels. In contrast, *VDR* mRNA expression in VAT was positively correlated with weight, BMI, glucose, and insulin. Moreover, the serum 25-OH-VD correlated negatively with *VDR* mRNA expression (*r* = −0.309, *p* = 0.027).

**Figure 1:**
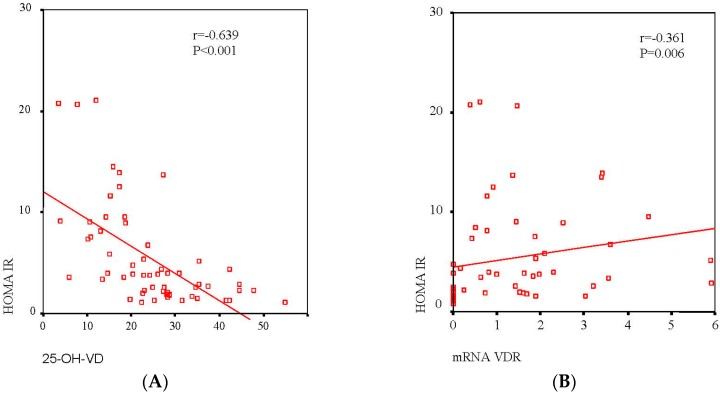
Association between serum levels of VD (**A**) and *VDR* mRNA expression in VAT (**B**) with HOMA-IR. A linear relationship was determined by Pearson’s correlation coefficient test; 95% confidence interval.

The correlation between serum 25-OH-VD and *VDR* mRNA in association with IR is shown in Figure 1. The serum 25-OH-VD level (*r* = 0.639, *p* < 0.001) correlated significantly with HOMA-IR (Figure 1A), and there was a negative correlation between mRNA *VDR* expression and HOMA-IR (*r* = 0.361, *p* = 0.006, respectively) (Figure 1B). However, to evaluate the independence of the association between HOMA-IR serum 25-OH-VD level and mRNA *VDR* expression, a multiple linear regression analysis was conducted as shown in Table 3. After inclusion of sex, age, BMI, waist circumference, 25-OH-VD, and *VDR* mRNA as independent variables, serum 25-OH-VD level remained significantly associated with HOMA IR.

### 2.3. VDR mRNA and Protein Level in Visceral Adipose Tissue

**Figure 2:**
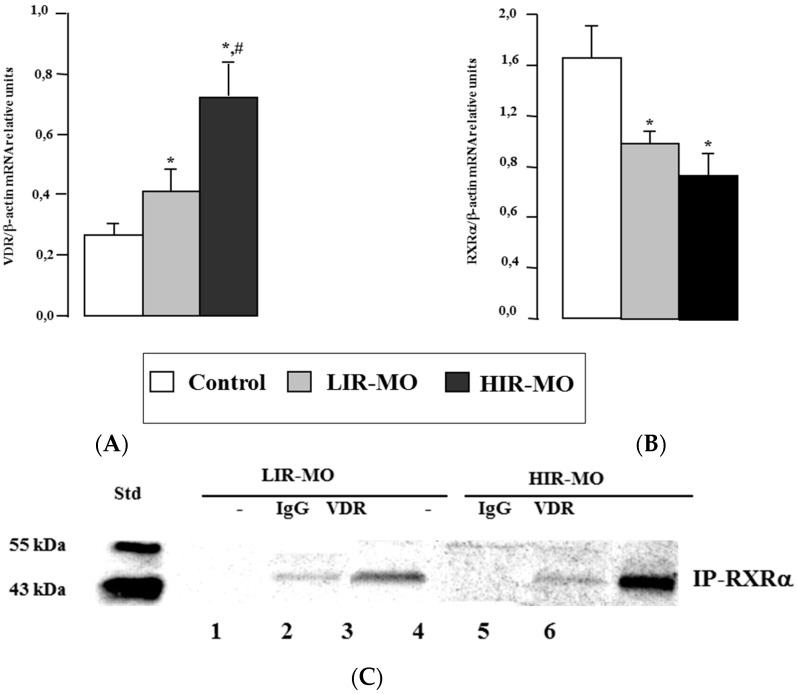
*VDR* (**A**) and *RXRa* (**B**) mRNA expression of VAT obtained from control, LIR-MO and HIR-MO subjects. The mRNA expression of these NRs was normalized to aP2 level. Results were expressed as the mean ± SEM and significance of differences * *p* < 0.05, between control vd MO groups, and # *p* < 0.01 between LIR-MO and HIR-MO.VDR coimmunoprecipitates with RXRa in nuclear extracts from VAT of the MO. Nuclear extracts of the VAT expressing VDR were preincubated with 1,25-(OH)2D3 (**C**) for 2 h before they were subjected to immunoprecipitation using rat antihuman RXR antibodies (lanes 3 and 6) or purified rat IgG (lanes 2 and 5) as a control. The VDR with RXRa in the protein complexes recovered by immunoprecipitation were detected by Western immunoblot (IB) analysis using rabbit antihuman VDR antibodies (**C**). Ten micrograms of the soluble nuclear (lanes 1 and 4) extracts were also analyzed to evaluate the relative abundance of the target proteins. IgG control, in lanes 2 and 5 shows the typical weak background signal. Nevertheless, we observe a stronger signal in lanes 3 and 6 (LIR-MO and HIR-MO, respectively) which is indicative of coimmunoprecpitation success. Moreover, there is a stronger signal for HIR-MO subjects than for LIR-MO. No signal was detected in the negative control (lanes 1 and 4) where no antibody was added.

In Figure 2, real-time PCR analysis showed that *VDR* mRNA expression in VAT was significantly higher in HIR-MO than in LIR-MO subjects and lean subjects (Figure 2A). In the case of its heterodimeric partner, *RXRa* mRNA expression was low in VAT from both MO groups in comparison with controls (Figure 2B). Finally, the expression levels of *VDR* mRNA in VAT increased significantly with the BMI and IR of the subjects, in contrast to 25-OH-VD.

To confirm the formation and presence of VDR heterodimerization with RXRa in VAT, we performed a series of co-immunoprecipitation experiments using nuclear extracts treated with 1,25(OH)_2_D_3_ (Figure 2C) for 4 h before co-immunoprecipitation. After VDR was immunoprecipated, a rat anti-human RXRa antibody was used to detect RXR bound to VDR. Positive detections were found in both groups of MO when using the VDR (lanes 3 and 6) but not in the IgG controls (lanes 2 and 5). These results were similar to mRNA expression, where it seems that VDR was higher in HIR-MO than in LIR-MO subjects. No protein bands corresponding to VDR were observed in the inmunoprecipitated complex with RXRa in the absence of the primary antibody against VDR (lanes 1 and 4).

### 2.4. DNA Binding Activities between VDR with RXRa in Human VAT

**Figure 3:**
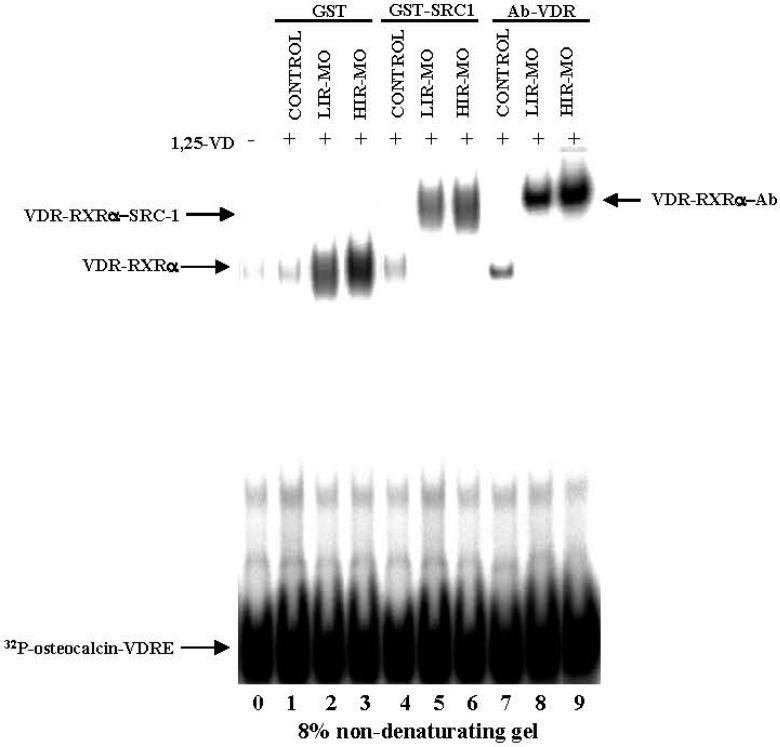
VDR binding activity in VAT by the EMSA radioactive method. Combined gel shift and supershift experiments were performed with equal amounts of nuclear extracts of VAT from control (lanes 1, 4, and 7), LIR-OM (lanes 2, 5, and 8) and HIR-OM (lanes 3, 6, and 8) to show the ability of VDR to bind to the ^32^P-labeled osteopontin VDRE sequence. Nuclear extracts were preincubated with VD as indicated in the panel A. Equal amounts of bacterially expressed wild-type GST (lanes 1, 2, and 3), GST-SRC1 (lanes 4, 5, and 6), and anti-VDR (lanes 7, 8, and 9) were added as indicated. Protein-DNA complexes were resolved from free probe through 8% non-denaturing polyacrylamide gels. Lane 10 of panel B represents the probe alone without nuclear extract, used as a negative control of the system.

To compare the ability of VDR to bind as a heterodimer with RXRa and to transactivate VDRE on DNA, we performed competitive EMSAs (Figure 3). Nuclear extracts prepared from 1,25(OH)_2_D_3_-treated VAT from the control and MO groups were incubated with a ^32^P-labelled VDRE (osteopontin DR3-type VDRE) sequence. As shown in Figure 3, one main specific shifted band was detected for the position belongs to the complex VDR–RXR heterodimers. This complex was markedly more abundant in HIR-MO subjects (lane 3) in comparison with the LIR-MO subjects (lane 2), and did not exist in the control group (lane 1). Furthermore, we analyzed the specificity of VDR-RXR heterodimer/coactivator interaction by performing EMSA in the presence of VD and coactivator (SRC-1). SRC-1 is able to stabilize the binding of VD-activated VDR-RXR heterodimer binding to VDRE in MO (lanes 5 and 6), but not in control subjects (lane 4).

Figure 3 also shows that when anti-VDR antibodies were included in the incubates of nuclear extracts with the [^32^P]VDRE, they were able to produce the most intense VDR supershift, which impaired VDR binding to DNA. The amount of [^32^P]VDRE supershifted by anti-VDR was also larger in HIR-MO subjects (lane 9) than in LIR-MO subjects (lane 8), no supershifted band formed in control subjects (lane 7). As a control, we used nuclear extracts with a 100-fold excess unlabeled primer, which resulted in a complete loss of signal (data not shown). Lane 0 of the gel represents the probe alone without nuclear extract, used as a negative control of the system.

### 2.5. IGFBPs mRNA and Protein Expression in VAT

**Figure 4:**
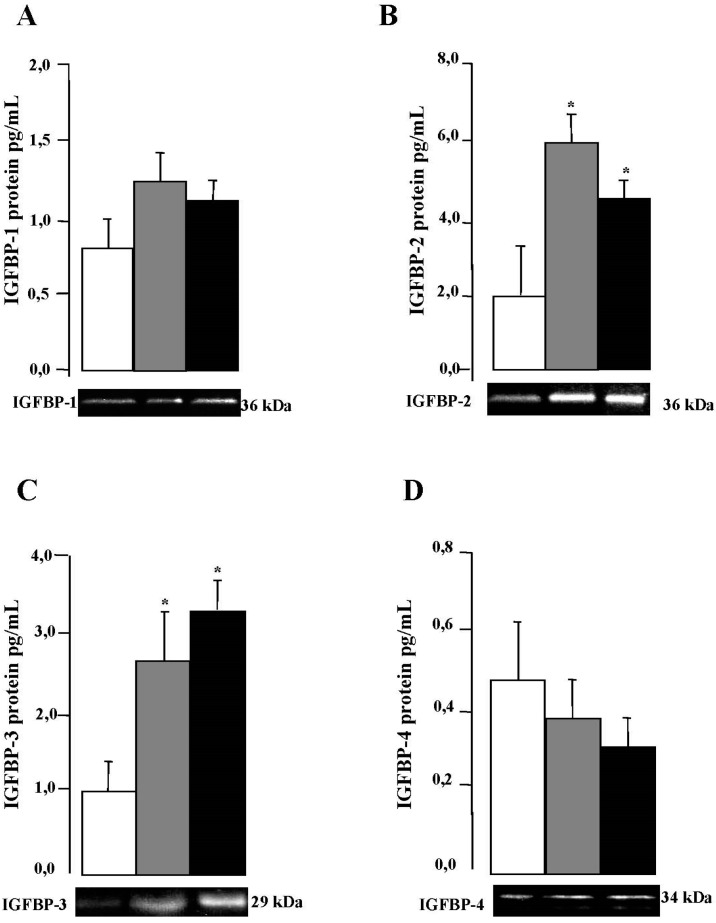
Significance of visceral adipose tissue IGFBP protein levels in control, LIR-MO and HIR-MO groups. xMAP assay of protein levels (columns in the “*y*” axis) and Western blotting (pictures in the “*x*” axis ) of IGFBP-1 (**A**); IGFBP-2 (**B**); IGFBP-3 (**C**); and IGFBP-4 (**D**) were done for each group. Significance of differences * *p* < 0.01 was compared with the control group. Columns represent the mean; bars represent SE.

In Table 4, we showed for the first time that *IGFBP-1*, *2*, *3*, and *4* are expressed by VAT form control and MO subjects, and the expression levels of *IGFBP-2* and *3* are higher in comparison with *IGFBP-1* and *IGFBP-4* in all the groups. Real-time PCR analysis showed that *IGFBP-2* and *IGFBP-3* mRNA expression are significantly higher in MO subjects in comparison with controls, but not in *IGFBP-1* and *IGFBP-4*. Next, we analyzed the quantitative determination of IGFBP-1–4 protein expression in VAT by Milliplex High Sensitivity Human IGFBP Immunoassay (Millipore Corporation, Billerica, MA, USA) (Figure 4), which showed a profile similar to that of gene expression with a significant decrease in the VAT depot in the control groups. IGFBP-2 protein level was higher in the LIR-MO subjects than in the HIR-MO subjects (Figure 4B). In contrast, IGFBP-3 protein expression was increased in the VAT depot of HIR-MO subjects in comparison with LIR-MO subjects (Figure 4C). However, we did not observe any differences in the quantification of IGFBP-1 (Figure 4A) and IGFBP-4 (Figure 4D) protein between LIR-MO subjects and HIR-MO subjects.

### 2.6. Expression and Activation of the Transcription Factor NF-κB

**Figure 5:**
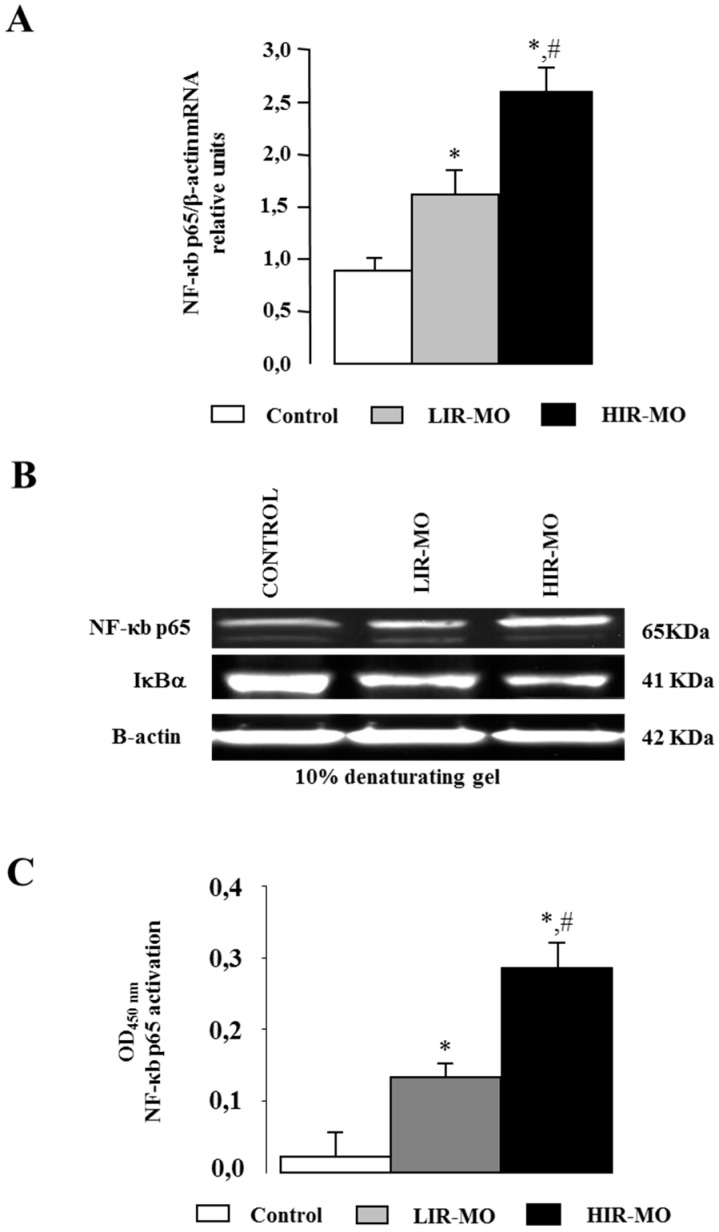
NF-κB activation in non-obese, LIR-MO, and HIR-MO. Quantitative RT-PCR for *NF-κB* (**A**) mRNA was performed on human VAT. Specific mRNAs were normalized to b-actin levels. The panels show the mean ± SEM from 8 subjects per group. Significant differences (Duncan, *p* < 0.05) are indicated with different letters. Total protein extracts were immunoblotted with antibodies targeting NF-κB *p*65, IκBα, and b-actin (**B**) and for detecting binding affinity of NF-κB *p*65 (**C**) families to the response element consensus oligonucleotide on 96-well plates (Trans-AM kits). Means (± SEM, *n* = 8). Significance of differences * *p* < 0.05, between control vd MO groups, and # *p* < 0.01 between LIR-MO and HIR-MO.

Different studies have shown an antagonist effect between VDR and NF-κB. To test the role of those transcription factors in the development of IR and obesity, we analyzed the mRNA and protein expression of NF-κB in the nuclear extracts of VAT from LIR-MO, HIR-MO and non-obese control groups (Figure 5). *NF-κB p65* gene expression was significantly increased in the HIR-MO group in comparison with the LIR-MO and control groups (Figure 5A). We next investigated the protein level by immunoblotting. Our results indicate that protein levels of NF-κB in control and MO subjects show results similar to those of mRNA expression levels (Figure 5B). In support of our results, protein levels of the Ikb-a subunit were higher in controls in comparison with MO patients (Figure 5B) implying that NF-κB is activated by IR in MO. These results were further supported by the confirmation of transcriptionally-active NF-κB *p*65 DNA binding, which was significantly increased in HIR-MO in comparison with LIR-MO subjects and controls (Figure 5C).

### 2.7. Identifcation of a Functional VDRE in the Promoter of the IGFBP-3 Gene by EMSA

**Figure 6:**
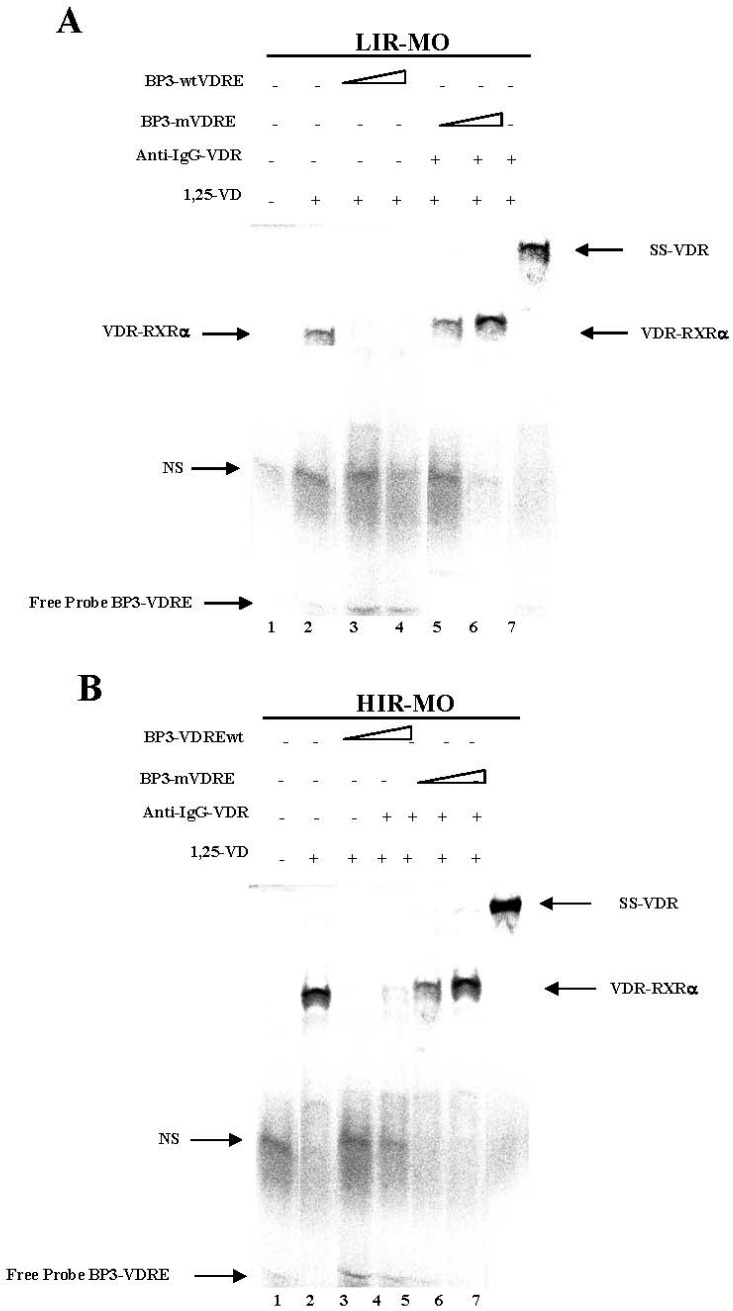
Specific binding activity of VDR to the putative BP3-VDRE sequences by the EMSA non-radioactive method. The biotin 3′ end labelled probe BP3-VDRE (**A**,**B**) was incubated with 5 µg of nuclear extract isolated from LIR-MO (**A**) and HIR-MO (**B**) both in the presence of 1,25-d (lane 2). For competition experiments, wild-type non-labelled BP3-wtVDRE (**A**,**B**) (lanes 3, 4, and 5), and mutated BP3-mVDRE (**A**) (lanes 5, 6, and 7). Supershift was performed with specific antisera to VDR (**A**,**B**) yielding supershifted complexes (lane 7). Lane 1, no nuclear extract.

We used EMSA to determine whether the VDR can bind to the putative IGFBP-3 VDRE sequence (Figure 6). The VDREs are generally composed for two direct repeats of six bases separated by a three-nucleotide spacer referred to as a DR3 motif. Thus, double-strand IGFBP-3-VDRE (BP3-VDR) (Figure 6A,B) oligonucleotides were incubated with nuclear extracts of VAT from LIR-MO (Figure 6A) and HIR-MO subjects (Figure 6B). No complexes were formed when nuclear extract was not added (Figure 6A,B, lane 1). A strong specific DNA-VDR-RXRa complex was observed with probe BP3-VDRE in HIR-MO in comparison with LIR-MO nuclear extracts (lanes 2). When a 500-fold molar excess of unlabeled oligonucleotide BP3-VDRE was added, the signal was diminished (lanes 3 and 4). In contrast, when an unlabeled oligonucleotide-containing mutation in the 5′ sequence (BP3-mVDRE) was added, DNA-binding was no longer competed (lanes 5 and 6). These results indicate that BP3-VDRE may be similar to the osteocalcin-VDRE that binds VDR with RXRa by radioactive EMSA assays. To confirm the presence of VDR and protein in BP3-VDRE, VDR antibody was added for supershift assays. As shown in Figure 6A,B, the BP3-VDRE sequence was bound by proteins recognized by VDR antibodies, thus producing a supershift complex SS-VDR, stronger in the HIR-MO (Figure 6B, lane 7) than in the LIR-MO nuclear extracts (Figure 6A, lane 7). No differences were found between groups in BP1-VDRE in the promoter nucleotide sequence of the *IGFBP-1* gene. No functional VDRE was found in the *IGFBP-4* promoter [23]. These results indicate a selective and specific binding of the VDR for *IGFBP-3* in MO subjects with different degrees of insulin resistance.

## 3. Discussion

Obesity is the most important known determinant for type 2 diabetes and is also an established risk factor for VD insufficiency among many populations worldwide [24]. Our findings confirm an inverse association between serum 25-OH-VD (a measure of vitamin D status) and BMI in the subjects studied. Emerging studies suggest a role of VD deficiency in the etiology of type 2 diabetes [25], although it has been argued that the influence of VD could be weak and that obesity may modify the association between 25-OH-VD and insulin sensitivity [26]. The data presented here demonstrate that subjects with obesity and a high degree of IR have a clear decline in serum 25-OH-VD. We also demonstrate a clear negative correlation between serum 25-OH-VD levels and increased HOMA-IR index. 

It might also be expected that VDR would be inverse with the BMI and the degree of IR., however, we showed a higher VDR protein and gene expression in MO patients, and we did not find any expression in the non-obese control group. We also found a positive correlation between VDR expression and the biochemical parameters associated with IR. It is conventional wisdom that 1,25(OH)_2_D_3_ hormone, a fat soluble agonist for the VDR, is stored in body fat, and to become metabolically active is first hydroxylated at position 25 of the sterol molecule. There is a practical limit to the 25-hydroxylation of VD, and when 1,25(OH)_2_D_3_ exceeds that limit, this hormone is accumulated within the body, both in serum and probably in body fat. However, in vivo proportion between the native compound and its derivatives 25-OH-D and 1,25(OH)_2_D_3_ at various inputs in humans is largely unknown, as are the kinetics of the conversion in vitro [27]. 

It has been demonstrated that the intracellular concentrations of 1,25(OH)_2_D_3_ can play an important role in either promoting or inhibiting adipogenesis (which may be a target for modulation in the treatment of obesity) via the VDR and transcriptional pathways that it targets [28]. Further examinations for this hypothesis in vivo may shed new light in the biology of the adipogenesis. For instance, for a better understanding of VD signaling in adipogenesis, the VDR expression in adipose tissue in subjects with different BMI and HOMA-IR should be studied. Surprisingly, our data demonstrate the opposite of what is to be expected, that subjects with higher BMI and degree of IR have a clear rise in gene and protein expression of VDR in VAT. However, it is also shown that the VDR carries out important biological activity in the absence of its ligand and has the ability to either inhibit or activate gene expression in the presence of its ligand [29]. The mechanism by which the unliganded and liganded VDR regulate adipogenesis will provide further insight into the role of the vitamin D signaling pathway in obesity. We also demonstrated, in co-immunoprecipitation studies with co-activators, that the interaction between VDR and RXRa is different in HI-MO and LIR-MO. Taking these results together with those on mRNA and protein expression, we can assume that VDR may have greater interaction with RXRa in the MO population in which it was more highly expressed. The mechanism by which the VDR acts is not clear, but the possibility exists that an adipocyte-specific cofactor mediates the effects of the unliganded VDR on lipid accumulation [30].

Interestingly, it has been described that VD decreases the chronic pro-inflamamtory status of adipose tissue by down-regulating pro-inflammatory cytokine production in a process in which NF-κB and VDR is involved [31]. In fact, we observed that NF-κB and *VDR* mRNA as well as protein expression are higher in HIR-MO patients, which could explain a new interaction in VAT which relates IR and MO.

Metabolic interaction has been reported between the VD and IGF-1 axes experimentally, with evidence to show that IGF-1 exerts some effects through changes in VD activation while 1,25(OH)_2_D_3_ in turn modulates the regulation of IGF-1 axis genes [16,17,18]. We showed a clear positive association between higher VD status and increased circulating IGF-1 concentration, an association independent of important putative confounders, such as lifestyle factors and adiposity. It is known that the concentration of serum levels of IGFB1, 2, and 3 change in obesity and IR [32]. However, there is insufficient data on the level of these binding proteins in adipose tissue. 

Our results showing higher expression of IGFBP-3, but not IGFBP-1, in VAT from the MO subjects are in accordance with other animal and in vitro studies [14]. Moreover, we showed that the expression of IGFBP-3 changed in both MO populations according to different degree of IR, despite being proteins that are very similar, structurally [33]. It is known that IGFBP-3 is involved in hyperglycemia, glucose intolerance, and IR, but these effects cannot be explained by circulating free IGF-1 levels [34]. This observation, together with the previous reports of nuclear localization of IGFBP-3 and interaction with RXRa, indicate that IGFBP-3 may play a role in modulating nuclear transcription of various genes of a variety of enzymes involved in glucose and lipid metabolism [35] that, in part, change the function of the fat cells to increase IR in humans. Our functional studies by EMSA suggested that the differential expression pattern of IGFBP-3 in obesity is regulated by higher expression of the VDR in HIR-MO and LIR-MO, respectively, in adipose tissue. The activation of VDR by decreased serum levels of 25-OH-VD in MO patients may be stimulated by the expression of IGFBP-3, which is highly involved in the development of IR.

In summary, our results indicate that serum levels of 25-OH-VD in MO subjects play a key role in the expression of VDR associated with IR. The regulation of IGFBP-3 by VDR activation may be one of the local and competitive mechanisms used by adipocytes to limit further fat gain and IR.

## 4. Material and Methods

### 4.1. Subjects

This study included 44 morbid obese (MO) subjects (defined by a BMI ≥ 40 kg/m^2^) and 15 lean subjects (defined by a BMI < 25 kg/m^2^). MO patients were classified in two groups according to insulin resistance (IR): non-diabetic MO with low IR (HOMA-IR: homeostasis model assessment of insulin resistance): MO with low IR (HOMA-IR < 5) (LIR-MO) and non-diabetic MO with high IR (HOMA-IR > 8) (HIR-MO) [36].

VAT biopsies were obtained from the epiplon of MO patients undergoing bariatric surgery procedures while VAT from lean subjects was obtained from mesenteric depot of patients whom had undergone laparoscopic surgery for elective cholecystectomy or hiatal-hernia surgery. Tissue samples were immediately frozen in liquid nitrogen and stored at −80 °C for the different assays described below. Patients were excluded if they had diabetes mellitus, cardiovascular disease, arthritis, acute inflammatory disease, infectious disease, renal disease, were receiving drugs that could alter the lipid or glucose profile, were under treatment with calcium or vitamin D supplements, or if they consumed >20 g ethanol per day at the time of inclusion in the study. The study was conducted according to the principles of the Declaration of Helsinki. All participants gave written informed consent, and the study was reviewed and approved by the Ethics and Research Committee of *Virgen de la Victoria* Hospital (Malaga, Spain).

### 4.2. Laboratory Measurements

Before surgery, and after an overnight fast, blood samples were obtained from the antecubital vein and placed in vacutainer tubes (BD vacutainer™). The serum was separated by centrifugation for 15 min at 4000 rpm and immediately frozen at −80 °C until analysis. Insulin was analyzed by an immunoradiometric assay (BioSource International, Camarillo, CA, USA) in a Beckman Coulter (Fullerton, CA, USA), showing 0.3% cross-reaction with proinsulin. The intra-assay and inter-assay CV was 1.9% and 6.3%, respectively. Serum glucose, cholesterol, triglycerides, HDL cholesterol (HDL-C), and C-reactive protein (CRP) were measured in a Dimension autoanalyzer (Dade Behring Inc., Deerfield, IL, USA) by enzymatic methods (Randox Laboratories Ltd., Barcelona, Spain). Glucose intra-assay CV was 7.5% while inter-assay CV was 13.5%. LDL cholesterol (LDL-C) was calculated using the Friedewald equation. Insulin was quantified by radioimmunoassay supplied by BioSource International Inc., Camarillo, CA, USA. HOMA-IR was calculated with the following equation: HOMA-IR = fasting insulin (µIU/mL) × fasting glucose (mmol/L)/22.5. Vitamind D or 25(OH)D levels were determined by enzyme immunoassay (ELISA) kits (Immundiagnostik AG, Bensheim, Germany), deficiency was considered if levels were <20 ng/mL (50 nmol/L). The inter-assay CV was 5.6% and the intra-assay CV was 7.3% [37].

### 4.3. VAT RNA Isolation and Real-Time Quantitative PCR

Total RNA isolation from VAT was obtained using RNeasy Lipid Tissue Mini Kit (Qiagen GmbH, Hilden, Germany). The purity of the RNA was determined by the 260/280 absorbance ratio on the Nanodrop. The integrity of total purified RNA was checked by denaturing agarose gel electrophoresis and ethidium bromide staining. For first-strand cDNA synthesis, a constant amount of 1 µg of total RNA was reverse transcribed using random hexamers as primers and Transcriptor Reverse Transcriptase (Roche, Mannheim, Germany). Gene expression was assessed by real-time PCR using an Applied Biosystems 7500 Fast Real-Time PCR System (Applied Biosystems, Darmstadt, Germany) with TaqMan technology, as previously described [37]. The commercially available and prevalidated TaqMan primer/probe sets used were the following: *VDR* (Hs01045840_m1, RefSeq. NM_000376.2, NM_001017535.1 and NM_001017536.1), *NF-κB* (Hs00765730_m1, RefSeq. NM_001165412.1 and NM_003998.3), *IGFBP-1* (NM_000596), *IGFBP-2* (NM_000597), *IGFBP-3* (NM_000598), *IGFPB-4* (NM_001552), *IGFBP-5* (NM_000599) and *PPIA* (4326316E, RefSeq. NM_021130.3) used as an endogenous control for each reaction. A threshold cycle (*C*t value) was obtained for each gene amplification curve and a ∆*C*t value was first calculated by subtracting the *C*t value for human *PPIA* cDNA from the *C*t value for each sample and transcript. Fold changes compared with the endogenous control were then determined by calculating 2^−Δ*C*t^, and expression results are expressed as the expression ratio relative to PPIA gene expression for humans, according to the manufacturer’s guidelines. All samples were quantified in triplicate, and positive and negative controls were included in all the reactions.

### 4.4. Western Blotting and Ligand-Immunoblot Analysis

Total protein extracts (T-PER, Active Motif, Belgium), cytoplasmic and nuclear extracts (N-PER Active Motif, Belgium) were prepared from VAT according to the manufacturer’s instructions. Protein concentration was determined using the Bradford method (Thermofisher Scientific Pierce Protein Biology, Waltham, MA, USA) and using bovine serum albumin as standard. Total protein extracts (30 μg) were separated by SDS-PAGE (sodium dodecyl sulfate polyacrylamide gel electrophoresis), blotted onto a PVDF membrane, and then incubated with specific antibodies against NF-κB and IGFBP proteins family (Santa Cruz Biotechnology-Sigma Antibodies, Heidleberg, Germany). After that, the membranes were incubated with the respective secondary anti-IgG antibodies (Santa Cruz Biotechnology-Sigma Antibodies). Protein signals were developed with Super-signal West-Pico Western blot detection kit (Thermofisher Scientific Pierce Protein Biology, Waltham, MA, USA), and were detected by electro-chemiluminescence detection Auto-Chemi-system analysis software Labworks 4.6 (UVP; Bio-Imaging Systems DBA Analytik Jena US).

To perform ligand-immunblot analysis, aliquots of adipose tissue were incubated with 1,25(OH)_2_D_3_ (10 μM) 4 h before the extraction of nuclear fractions. The nuclear extracts (100 μg) were precleared by incubation in IPP buffer (20 mM HEPES (pH 7.5), 20% glycerol, 0.1% Nonidet P-40 and protease inhibitors (complete protease inhibitor cocktail; Roche Diagnostics, Indianapolis, IN, USA)) and 25 μL protein G plus/protein A-agarose (Sigma) for 1 h at 4 °C with gentle rotation. The pre-cleared lysates were collected and the nuclear receptors VDR were co-precipitated by incubating with 2 μg rat antihuman RXR antibody (Santa Cruz) or purified rat IgG (Sigma) for 20 h at 4 °C with gentle rotation. Protein G plus/protein A-agarose (25 µL) was added, and the incubation continued for 1 h at romm temperature to allow the capture of the antibody-protein complexes by the agarose beads. The immune-precipitated protein complexes were washed in buffer containing 20 mM HEPES (pH 7.5), 75 mM KCl, 2.5 mM MgCl_2_, 0.1% Nonidet P-40, and protease inhibitors, recovered from the agarose beads and separated on SDS-PAGE. A mouse monoclonal anti-VDR (sc-13133) was used as primary anti-body and a goat anti-mouse IgG-HRP (sc-2005) both from Santa Cruz Biotechnology-Sigma Antibodies. Clarity Western ECL substrate (Bio-Rad, Hercules, CA, USA) were used for detection. All experiments were performed in duplicate.

### 4.5. Cytokine Determination

Of the total extract, 50–100 μg were obtained from VAT with a commercially-available kit (T-PER total protein Extraction Kit, Active Motif, Belgium). The Milliplex High Sensitivity Human IGF Binding protein (IGFBP) panel (cat NB0 HIGFBP-53k Millipore Corporation) according to the manufacturers’ protocols determined the quantitative determination of IGFBP-1, 2, 3, and 4. Multi-analyte profiling was performed on the Luminex-100 XMAP™ Technology (Luminex Corporation, Austin, TX, USA). The xMAP technology (Luminex Corporation), combines the principle of a sandwich immunoassay with fluorescent bead–based technology, allowing individual and multiplex analysis in a single microtiter well [38]. Acquired fluorescence data were analyzed by the Luminex 100 xMAP software 2.2 (Luminex Corporation).

### 4.6. Electrophoretic Mobility Shift Assay (EMSA)

Gel shift assays were performed with 25–50 mg of nuclear extracts from VAT. The proteins were pre-incubated on ice for 15 min in a total volume of 20 µL binding buffer (10 mM HEPES (pH 7.9), 150 mM KCl, 1 mM DTT, 0.2 µg/µL poly (dI-C), and 5% glycerol) [39]. For supershift assays, 1 μL of an antibody directed against VDR antibody (Santa Cruz Biotechnology-Sigma Antibodies)or 5–10 µg of a bacterial expressed GST-SRC-1_597-791_ (or GST alone as a negative control) were added to the reaction mixture before label probe [40]. Approximately 1 ng of the [P]-labeled32 double-strand oligonucleotides (50,000 cpm) corresponding to one copy of the human osteocalcin promoter DR6-type VDRE (core sequence: 5′-TTTGGTGACTCACCGGGTGAAC-3′) [41] was then added and incubated for 15 min at room temperature. Protein-DNA complexes were resolved by electrophoresis through 8% non-denaturing polyacrylamide gels in 0.5× TBE (45 mM Tris, 45 mM boric acid, 1 mM EDTA (pH 8.3). The gels were dried and quantified on a Fuji FLA3000 reader using Image Gauge software.

Nuclear extracts were tested for VDR-binding activity with promoter of IGFBPs consensus oligonucleotide for activity, employing consensus oligonucleotide for VDRE using the LightShift chemiluminescent EMSA kit (Pierce). Biotin 3′ end-labeled probes (Eurogentec) were prepared by annealing oligonucleotides. The oligonucleotides included hIGFBP-1 VDRE (5′-TGTGACCCCTGCCAGGGGGCAGT-3′) [42] and hIGFBP-3 VDRE (5′-AAATGCACCCGGTGAACCTCTC-3′) [27]. The DNA-protein binding assay was performed at room temperature for 20 min in a final volume of 20 µL. The reaction mixture was then subjected to gel electrophoresis on a 5–10% native polyacrylamide gel with Tris-Borate EDTA (TBE) buffer 0.5× and was transferred to a nylon membrane (Pierce). DNA-protein complex was fixed to the membrane by UV and detected by a nonradioactive nucleic acid detection kit (Pierce). For competition studies, DNA binding reaction mixtures were pre-incubated with unlabeled ds DNA oligos of the wild-type response elements or mutant sequences. For super-shift assays, 1 µL of an antibody directed against mouse monoclonal anti-VDR (sc 13133, Santa Cruz Biotechnology) were pre-incubated in the binding reaction for 10 min before the probe was added.

### 4.7. ELISA-Based TransAM™ Kit for p65 NF-κB

Nuclear extracts were prepared with a commercially-available kit (Nuclear Extract Kit, Active Motif), according to manufacturer’s instructions. After protein quantification with a Coomassie Plus assay kit (Pierce), nuclear extracts were checked for NF-κB (*p*50) activation using ELISA-based kits (TransAM™). Briefly, 5 µg of nuclear protein was added in a volume of 20 µL to a well plate precoated with oligonucleotides (5′-GGGACTTTCC-3′) corresponding to the NF-κB response element. Wells were then incubated with a primary antibody against NF-κB (*p*50) (1:1000) for 1 h. After washing, the plate was incubated with horseradish peroxidase-conjugated secondary antibody. The quantity of NF-κB (*p*50) proteins in nuclear extracts were quantified by measuring the absorbance at 450 nm on a Versamax™ microplate reader.

### 4.8. Statistical Analysis

Comparisons between controls and MO patients were made using Mann-Whitney *U*-test for non-parametrical variables. Comparisons between the three groups (LIR-MO, HIR-MO, control patients) were conducted using the Kruskal-Wallis test for non-parametrical variables. Spearman’s correlation coefficients were calculated to estimate the linear correlations between variables. The Mann-Whitney rank sum test was used to identify the significant differences in the median for each marker expressed among LIR-MO, HIR-MO and control groups. The results were given as the mean ± SD. SPSS statistical software, version 15.0 for Windows (SPSS Inc., Chicago, IL, USA), was used for the statistical analyses. Values were considered to be statistically significant when *p* ≤ 0.05.

## Figures and Tables

**Table 1:** Anthropometric and biochemical variables in control (lean) subjects, low-insulin resistance morbidly obese low insulin resistance (LIR-MO) patients, and high-insulin resistance morbidly obese (HIR-MO) patients.

Variables	Control	LIR-MO	HIR-MO
*N* (men/women)	8/7	5/6	5/7
Age (years)	47.0 ± 15.6 ^a^	45.6 ± 11.7 ^ab^	37.8 ± 9.6 ^b^
Weight (kg)	66.2 ± 11.8 ^a^	150.0 ± 26.9 ^b^	156.2 ± 18.7 ^b^
BMI (kg/m^2^)	23.1 ± 2.45 ^a^	56.5 ± 7.1 ^b^	55.4 ± 3.9 ^b^
Waist circumference (cm)	83.3 ± 10.5 ^a^	141.0 ± 16.3 ^b^	143.7 ± 20.2 ^b^
Glucose (mg/dL)	84.6 ± 14.7 ^a^	93.0 ± 10.1 ^b^	102.3 ± 10.9 ^b^
Insulin (μIU/mL)	9.2 ± 3.9 ^a^	14.1 ± 4.0 ^a^	44.5 ± 7.8 ^b^
Uric Acid (mg/dL)	4.28 ± 1.48	5.29 ± 0.98	6.04 ± 1.06
Cholesterol (mg/dL)	193.2 ± 44.0	204.5 ± 39.8	200.4 ± 23.5
HDL-C (mg/dL)	55.5 ± 17.2	46.8 ± 14.8 ^ab^	42.2 ± 17.9 ^b^
Triglycerides (mg/dL)	90.3 ± 50.4	111.3 ± 35.2	136.4 ± 91.2
AST	26.1 ± 17.7	19.6 ± 7.0	20.7 ± 3.8
ALT	46.3 ± 31.7	36.0 ± 12.6	49.5 ± 12.1
GGT	110.2 ± 341.1	51.4 ± 58.0	40.4 ± 14.0
HOMA-IR	1.99 ± 0.098 ^a^	3.27 ± 0.94 ^b^	11.28 ± 2.43
CRP (mg·L^−1^)	2.93 ± 3.66	2.74 ± 1.10	5.44 ± 4.73
Serum 25-OH-VD	34.6 ± 9.6 ^a^	20.5 ± 8.6 ^b^	16.3 ± 2.1

The results are given as the mean ± SD. Different letters indicate significant differences between the means of the different groups of subjects (*p* < 0.05; a: controls vs. MO groups, b: LIR-MO vs. HIR-MO) according to Student’s *t*-test and chi-squared test for variables expressed as a percentage. Body mass index (BMI); high density lipoprotein cholesterol (HDL-C).

**Table 2:** Spearman correlations between serum level of 25-OH-VD and expression of *VDR* with anthropometric and biochemical parameters in all the patients (control, LIR-MO, and HIR-MO patients).

Variables	25-OH-VD	mRNA *VDR*
Weight	−0.598 (<0.001)	0.448 (<0.001)
BMI	−0 637 (<0.001)	0.513 (<0.001)
Waist circumference	−0.589 (<0.001)	−0.517 (<0.001)
Glucose	−0.572 (<0.001)	0.361 (0.005)
Insulin	−0.602 (<0.001)	0.535 (<0.001)
HOMA-IR	−0.639 (<0.001)	0.499 (<0.001)

LIR-MO: non-diabetic morbidly obese with low IR; HIR-MO: non-diabetic morbidly obese with high IR; VDR: vitamin D receptor; BMI: body mass index.

**Table 3:** Multiple regression analysis where the dependent variable is the level of HOMA-IR and independent variables are sex, age, BMI, waist, 25-OH-VD, and mRNA *VDR* expression in VAT.

Variable	B	B_SD_	β	*p*
Sex	1.006	1.757	0.092	0.571
Age	−0.009	0.055	−0.021	0.864
BMI	0.216	0.180	0.598	0.239
Waist circumference (cm)	−0.047	0.080	−0.235	0.560
25-OH-VD	−0.186	0.080	−0.374	0.026
mRNA *VDR*	−0.118	0.077	−0.196	0.135
Constant	7.204	9.947		0.474

B and B_SD_: The unstandardized coefficients are the coefficients of the estimated regression model. β: The standardized coefficients or b are an attempt to make the regression coefficients more comparable. *R*^2^: 0.550. Significance of differences *p* < 0.05. VDR: vitamin D receptor; VAT: visceral adipose tissue; BMI: body mass index.

**Table 4:** Analysis of the mRNA IGFBP (1, 2, 3 and 4) expression in VAT from the control and morbidly obese groups (LIR-MO and HIR-MO).

Gene	Control	LIR-MO	HIR-MO
*IGFBP-1*	5.61 ± 1.28 ^a^	6.72 ± 2.21 ^ab^	6.85 ± 2.39 ^ab^
*IGFBP-2*	6.44 ± 1.11 ^a^	11.29 ± 2.03 ^ab^	10.03 ± 1.88 ^b^
*IGFBP-3*	7.46 ± 1.08 ^a^	10.03 ± 1.88 ^b^	11.20 ± 1.60 ^b^
*IGFBP-4*	4.23 ± 1.67 ^a^	2.56 ± 1.42 ^ab^	2.20 ± 1.03 ^ab^

The results are given as the mean ± SD. Different letters indicate significant differences between the means of the different groups (a: controls vs. MO groups, b: LIR-MO vs. HIR-MO) (*p* < 0.01). LIR-MO: non-diabetic morbidly obese with low IR; HIR-MO: non-diabetic morbidly obese with high IR.
